# Extensively Drug-Resistant New Delhi Metallo-β-Lactamase–Encoding Bacteria in the Environment, Dhaka, Bangladesh, 2012

**DOI:** 10.3201/eid2106.141578

**Published:** 2015-06

**Authors:** Mark A. Toleman, Joachim J. Bugert, Syed A. Nizam

**Affiliations:** Cardiff University, Heath Park Campus, Cardiff, Wales, UK

**Keywords:** New Delhi metallo-β-lactamase, NDM, multilocus sequence typing, sequence type, ST101, ST405, ST648, CTX-M-15, extensively drug resistant, bacteria, Bangladesh, environment, Escherichia coli, environmental water, sewage

## Abstract

Carriage of the New Delhi metallo-β-lactamase variant 1 (NDM-1) enables drug resistance to move between communities and hospitals. In Bangladesh, we found the *bla*_NDM-1_ gene in 62% of environmental waters and in fermentative and nonfermentative gram-negative bacteria. *Escherichia coli* sequence type (ST) 101 was most commonly found, reflecting a common global relationship between ST101 and NDM-1.

Carbapenemases, bacterial enzymes that typically inactivate most of the β-lactam class of antimicrobial drugs, have emerged rapidly over the past decade (*1*). These resistance mechanisms are often accompanied by other resistance alleles, and together they can confer extensive drug resistance, leaving minimal treatment options (*2*). The New Delhi metallo-β-lactamase variant 1 (NDM-1), a chimera formed by the fusion of 2 resistance genes, is unique among the carbapenemases (*3*). Since its description in 2009, NDM-1 has spread rapidly to many countries worldwide and appears to be endemic in South Asia (*1*,*4*,*5*). A study of the environment in New Delhi, India, showed that ≈30% of surface waters and sewage was contaminated with NDM-1; the enzyme was also detected in drinking water (*6*). In addition, high rates of NDM-1 gut carriage have been found in the community and in hospitals in Pakistan (*7*). High rates of gut carriage can lead to contamination of drinking water and food through inadequate sewage treatment. Furthermore, gut carriage of NDM-1–encoding *Escherichia coli* can lead to common community-acquired infections (e.g., urinary tract infections), which often require hospitalization (*8*) and enable resistance mechanisms to move between community and hospital sectors. Indirect studies in 2009 and 2010 showed that NDM-1 was not present in the Bangladesh environment (*9*,*10*). To determine whether NDM-1 is now present in Bangladesh, we surveyed the environmental waters of Dhaka. 

## The Study

**Figure 1:**
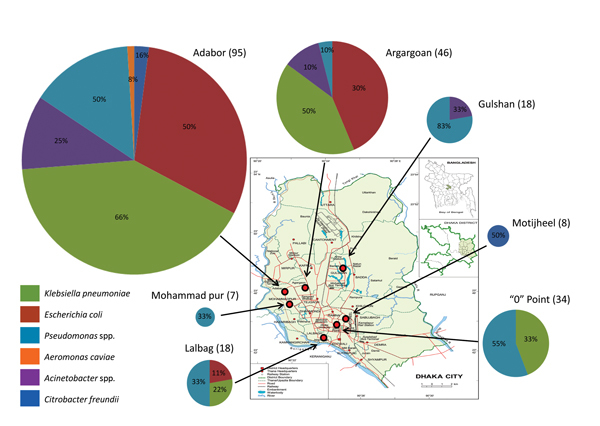
Diversity of New-Delhi metallo-β-lactamase variant 1–encoding species and the number found in 58 locations in 7 regions (red circles on map) of Dhaka, Bangladesh, October 2012. Individual sampling sites were within 2 km of each sampling region, and the number of sites varied from 6 to 12 per region. Pie charts indicate the proportions of different *bla*_NDM-1_–positive bacteria isolated in each region; colors indicate specific species. The diameter of each pie chart is directly proportional to the number of *bla*_NDM-1_–positive isolates collected in each region; actual numbers are shown in parentheses after the region name. Numbers within pie charts indicate the percentage of sites in each region in which the individual positive *bla*_NDM-1_–positive species were found. *bla*_NDM-1_ was detected in samples from all 7 regions and from 36 (62%) of the 58 sampling sites.

During October 19–27, 2012, we collected environmental water/sewage samples from 7 regions (58 sites) in Dhaka, Bangladesh (Figure 1). Control samples were from the United Kingdom. Each sample was investigated for bacterial growth on UTI brilliance agar plates (Thermo Fischer Scientific, Basingstoke, UK) containing vancomycin (30 mg/L) plus meropenem (0.5 mg/L). The species of individual colonies of different colors and morphologies were determined by using matrix-assisted laser desorption/ionization time-of-flight mass spectrometry. Bacteria were genetically characterized by *bla*_NDM-_specific PCR. Genetic location of the *bla*_NDM-1_ gene was determined by probing S1 nuclease pulsed-field gels. A subset of isolates of each species was further investigated for MICs of relevant antimicrobial drugs. All *E. coli* isolates were genotyped to determine multilocus sequence typing group; examples of each group were characterized for additional relevant resistance mechanisms. Details are provided in the Technical Appendix.

The carbapenemase and extended-spectrum β-lactamase genes *bla*_NDM-1_ and *bla*_CTX-M-15_ were detected by PCR in 36 (62%) and 41 (71%), respectively, of the 58 water samples. Both genes were found at all 7 sample region sites in Dhaka. Gene *bla*_CTX-M-15_, but not *bla*_NDM-1_, was detected in sewage samples from the United Kingdom; neither was detected in UK water samples from the River Thames.

We identified 226 gram-negative NDM-1–producing isolates to the species level (Figure 1; Technical Appendix Table 1); 15 isolates harboring *bla*_NDM-1_ could not be identified and were not investigated further. The most widely disseminated bacteria in samples from Dhaka were pseudomonads (6/7 regions) and *Klebsiella pneumoniae* (4/7 regions). Nine different species of *Pseudomonas* spp. and 5 *Acinetobacter* spp., mostly belonging to nonpathogenic strains, were among the nonfermentative bacteria (Technical Appendix Table 1). Carbapenem resistance in the *Pseudomonas* spp. isolates was unstable; all strains lost the *bla*_NDM-1_ gene after 2 days’ growth or when frozen for storage.

With the exception of 4 isolates, all bacterial isolates contained the original *bla*_NDM-1_ allele; 3 *E. coli* sequence type (ST) 101 isolates carried the *bla*_NDM-3_ variant, and 1 ST648 isolate carried the *bla*_NDM-4_ variant (Technical Appendix Table 2). S1 nuclease pulsed-field gel electrophoresis combined with *bla*_NDM-1_ probes detected *bla*_NDM-1_ on plasmids of limited size diversity in *E. coli* (ST101, 160 kb; ST405, 100 kb; ST648, 150 kb); however, other species included *bla*_NDM-1_–positive plasmids in a wide diversity of sizes (30 kb–450 kb); some of these species had multiple positive plasmids, and *bla*_NDM-1_ was also found on the chromosome (Technical Appendix Table 1 and Figure 1).

The *E. coli* isolates were further analyzed by PCR to identify additional resistance mechanisms often associated with *bla*_NDM-1_ (Table). *bla*_CTX-M-15_ and 16s ribosomal methylase genes (*armA* or *rmtB*) were associated with most *E. coli* strains, which explains the extensively drug-resistant phenotype of the *E. coli* isolates (Technical Appendix Table 3). Plasmids of plasmid incompatibility groups *inc*FII (ST101, ST405, ST648) and *inc*X (ST405, ST648) were also closely associated with *E. coli* strains (Table). *E. coli* harboring *bla*_NDM-1_ were isolated from 10 sampling sites (Figure 1; Technical Appendix Table 1). *The E. coli* isolates belonged to 3 different multilocus sequence typing groups: ST101 (phylogroup B1, 20/53 samples); ST405 (phylogroup D, 5/53 samples); and ST648 (phylogroup D, 28/53 samples) (Technical Appendix Table 2). ST101, which was found in samples from 6 (10.3%) of the 58 sites, was the most prevalent NDM-1–encoding *E. coli* genotype. ST648 represented an intermediate prevalence (5/58 [8.6%] sites), and ST405 was the least prevalent (1/58 [1.7%] sites) (Technical Appendix Tables 1, 2).

**Table:** Resistance genes and plasmid profiles for a subset of *Escherichia coli* strains in a study of extensively drug-resistant New Delhi metallo-β-lactamase–encoding bacteria in the environment, Dhaka, Bangladesh, October 2012*

***E. coli* strain, ST**	**Resistance genes**								
	*bla*_CTX-M-15_	*bla*_NDM_	16S methylase	*bla*_ampC_	*inc*X	*inc*FII	*inc*L/M	*inc*A/C	*inc*N2
**18, ST101**	+	NDM-3	*rmtB*	−	−	+	−	−	−
**24, ST101**	+	NDM-3	*rmtB*	−	−	+	−	−	−
**25, ST101**	+	+	*rmtB*	−	−	+	−	−	−
**28, ST101**	+	NDM-3	*rmtB*	−	−	+	−	−	−
**221, ST101**	+	NDM-1	*rmtB*	−	−	+	−	−	−
**34, ST648**	+	+	*armA*	*cmy*, *dha*	+	+	−	−	−
**192, ST648**	+	NDM-4	*armA*	*cmy*, *dha*	+	+	−	−	−
**346, ST648**	+	+	*armA*	*cmy*, *dha*	+	+	−	−	−
**43, ST405**	+	NDM-1	*armA*	*cmy*, *dha*	+	+	−	−	−
**54, ST405**	+	NDM-1	*armA*	*cmy*, *dha*	+	+	−	−	−

*armA and *rmtB*, aminoglycoside methylase genes; CTX-M-15, *cmy*, and *dha,* β-lactamases; *inc*, plasmid incompatibility group; NDM, New-Delhi metallo-β-lactamase; ST, sequence type; −, negative; +, positive.

## Conclusions

Our findings indicate that NDM-1 is widespread in the Dhaka environment. We detected 241 NDM-1–encoding bacterial isolates; they were found in all 7 sampled regions and at 36 (62%) of the 58 sampling sites. This high level of environmental *bla*_NDM-1_ contamination is of concern, especially because drinking water in Bangladesh usually carries high levels of sewage-derived bacteria (*11*). It is therefore likely that *bla*_NDM-1_ carriage rates will rise rapidly. Future environmental studies could provide indicators of epidemics of emerging resistant bacteria before they are realized in hospitals.

Despite the widespread presence of NDM-1 in Dhaka, it appears that this carbapenemase has recently emerged in the Bangladesh environment. Studies in northern Bangladesh did not find NDM-1 in wild ducks and poultry in 2009 (*9*) or in crow and gull feces in 2010 (*10*). Similarly, NDM-1 was not detected in drinking water in Dhaka during 2008–2009 (*11*) even though all samples had high levels of fecal and *bla*_CTX-M-15_ contamination. Furthermore, a study of 1,879 clinical *E. coli* and *Shigella* spp. isolates collected during 2009–2010 in Bangladesh did not detect *bla*_NDM-1_ (*12*). The first known clinical isolates date from 2008 (*12*), and the first evidence of human gut carriage of *bla*_NDM-1_ was found in samples collected in Dhaka (*13*) a month before our study.

**Figure 2:**
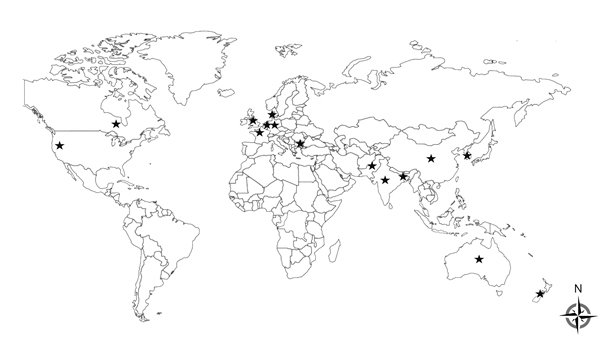
Sites where New-Delhi metallo-β-lactamase variant 1 (NDM-1)–encoding *Escherichia coli* sequence type (ST) 101 isolates have been detected worldwide. Stars indicate countries where NDM-encoding *E. coli* ST101 has been detected: Australia, Bangladesh, Belgium, Bulgaria, China, Canada, Denmark, France, Germany, India, Korea, New Zealand, Pakistan, the United Kingdom, and the United States.

Because *E. coli* is the leading cause of human urinary tract infections, bloodstream infections, and neonatal meningitis, the ability of NDM-1 to give this bacterium clinical resistance to carbapenems is of concern (*14*). *E. coli* is also universally carried in the human gut. Therefore, we focused on this species because it is likely to be the greatest threat to human health. *E. coli* encoding NDM-1 were found in 3 of the 7 sampled regions, and genotyping showed they belonged to only 3 STs: ST648, ST101, and ST405. These same 3 *E. coli* genotypes are responsible for 80% of clinical NDM-1–encoding *E. coli* isolates in the United Kingdom (*15*). Furthermore, ST101 is the most common *E. coli* genotype in the Bangladesh environment (10.3% prevalence) and in clinical isolates from the United Kingdom (50%). Results of a literature search for NDM-1–encoding *E. coli* belonging to ST101 showed that this genotype has been detected in 15 nations (Figure 2). Thus, *E. coli* ST101 appears to be a successful global genotype that is often associated with NDM-1. This association with a single global genotype is analogous to the association between *E. coli* ST131 and the cephalosporinase CTX-M-15. Because of the critical nature of extensively drug-resistant bacteria, we are investigating the underlying factors responsible for the success of these particular antimicrobial drug–resistant strains.

**Technical Appendix.** Methods and Results for a study of extensively drug-resistant New Delhi metallo-β-lactamase–encoding bacteria in the environment, Dhaka, Bangladesh, October 2012; genomic location of *bla*_NDM-1_ in environmental bacteria from Dhaka; list of isolates; and phylogenetic analysis of NDM-positive *Escherichia coli* from the environment
