# Supplementary material for: Extensively Drug-Resistant New Delhi Metallo-β-Lactamase–Encoding Bacteria in the Environment, Dhaka, Bangladesh, 2012
Source: Emerg Infect Dis. 2015 Jun;21(6):1027–30. doi: 10.3201/eid2106.141578 (PMC4451920; doi:10.3201/eid2106.141578)
Supplement: Supplementary file 1 — Technical Appendix. Methods and Results for a study of extensively drug-resistant New Delhi metallo-β-lactamase–encoding bacteria in the environment, Dhaka, Bangladesh, October 2012; genomic location of blaNDM-1 in environmental bacteria from Dhaka; list of isolates; and phylogenetic analysis of NDM-positive Escherichia coli from the environment [file 14-1578-Techapp-s1.pdf]

Article DOI: http://dx.doi.org/10.3201/eid2106.141578

Extensively Drug-Resistant New Delhi Metallo-β-Lactamase–Encoding Bacteria in the Environment, Bangladesh, October 2012

Technical Appendix

Supplemental Methods

Sample Collection and Transport

The samples included surface waters, water from a lake, gray water from residences and sewage samples all from Dhaka city, Bangladesh. Sample sites included the Motijheel, Agargaon, Gulshan, Adabor, Lalbag, "0" point and Mohammad Pur areas of Dhaka city and included 8-10 samples within a 2 kilometer radius of each location. Each sample consisted of about 100 µl of liquid absorbed onto a sterile charcoal swab and was collected by dipping the sterile swab in the water source. Samples were transported in UN 3373 approved sealed containers by personal courier to Cardiff, UK. Control water samples were collected from sewage processing plants in South Wales (Nash, Ponthir, Weycock, Coslech, Cogmoors, Creigau, New Drope, Cowbridge, East Bon and Rhydlafar) and the river Thames. All sewage samples from the UK were influent samples before any sewage processing had taken place.

Initial Gene Detection

The NDM-1 gene (bla_{NDM-1}) and the CTX-M-15 gene ($bla_{CTX-M-15}$) were initially detected by PCR directly from the swab using 1µl of sample water squeezed from the swab as template and standard primers and conditions.

Bacterial Selection, Identification and MIC Determination

Each swab was streaked on UTI brilliance agar plates containing vancomycin (30 mg/L) plus meropenem (0.5 mg/L) and grown overnight at 37°C. Following incubation, bacterial growth was collected by sweeping a sterile loop several times across the plate ensuring that colonies of many different colors and morphologies were collected. The bacterial culture was

transferred to 0.5 ml of sterile water in an Eppendorf tube and resuspended. One µl of this bacterial suspension was then used for PCR reactions to detect the NDM-1 gene (*bla*$_{\text{NDM-1}}$) and the CTX-M-15 gene (*bla*$_{\text{CTX-M-15}}$) and the rest stored at 4°C. Samples that were positive for *bla*$_{\text{NDM-1}}$ in the initial PCR were then re-plated at several dilutions to ensure well-spaced colonies. From these plates at least twenty further well-spaced colonies were re-grown and individually investigated by PCR and sequencing for the presence of *bla*$_{\text{NDM-1}}$. These represented a range of colonies of different color and morphology to ensure detection of as many different bacterial NDM-1 hosts at each site as possible.

Positive individual cultures were again streaked on brilliance agar plates to confirm purity and then speciated by Maldi-TOF using the Bruker MALDI Biotyper system, Mcroflex LT and Biotyper 3.0 software (Bruker Daltonics, Germany) and NDM presence re-confirmed by PCR. Isolates of each species were tested for MICs against several antibiotics (ETest, bioMérieux, Basingstoke, UK. Liofilchem, Roseto degli Abruzzi, Italy) on Mueller-Hinton plates (Becton Dickinson, Oxford, UK) and the results determined according to the manufacturer's instructions.

NDM Allele

Approximately 10 positive PCR products from each genus including duplicate examples from each individual species were sequenced to confirm the accuracy of the PCR reaction and also to determine the NDM variant present (these were amplified using primers NDMVF-TGGCTTTTGAAACTGTCGCACC and NDMVR-CTGTCACATCGAAATCGCGCGA designed up and downstream of the gene to ensure the entire gene was sequenced).

Gene Location

The location of the *bla*$_{\text{NDM-1}}$ gene in each bacterial isolate was determined by pulsed field gel electrophoresis (PFGE) following S1 digestion of macro DNA in agarose plugs. The genomic location was detected using in-gel hybridization with a P^{32}-labeled *bla*$_{\text{NDM-1}}$ probe at 65°C as described previously.

Additional Resistance Alleles

E. coli bla$_{\text{NDM-1}}$ positive isolates (10) collected from a range of sites that were positive for these species were further investigated for additional resistance alleles that are often found

associated with *bla*$_{NDM-1}$ including the 16S ribosomal methylase genes *armA, rmtB, rmtC, rmtF, ampC* genes and *bla*$_{CTX-M}$ genes by multiplex PCR using published primers and conditions.

Common NDM Plasmid Types

Plasmid types that are commonly associated with *bla*$_{NDM-1}$ were investigated by PCR in the above *E. coli* isolates using the custom designed primer pairs itemized below. These were designed to amplify plasmid backbone genes from NDM harboring plasmids available in the genetic data bases and amplified using relevant controls.

*inc*N2 F/R-ACTCACGTTCGCCTGGATTT and GCCACCCTTAACCTGTTCGA; JQ349085

*inc*FII F/R-TGCAGAGTTCGCTGCCGGTG and TACGCCCGGCATCTCCCACA; HG003695

*inc*L/M F/R GGTCAGCACCGTTGACCGGG and CGTCTTTCGGCAGCGTCCGT; JX988621

*inc*X F/R ATGGGCGCATCTTTTTGCGAAGGA and TTTCCTGTCGCTCAGGACTTCA; JX104760

*inc*AC F/R GAGAACCAAAGACAAAGACCTGGA and TTCTGGAGTTCGTACAGAGTGAAC. JF503991

E. coli Phylogeny

The 53 *bla*$_{NDM-1}$ positive *E. coli* isolates collected in this study were further investigated for phylogenetic grouping using the recently updated (2013) Clermont multiplex PCR method and also by the high resolution 2 loci clonal EXPEC typing method, using the described primers and conditions and applicable sequencing. Several examples of each different group determined by these methods were also subjected to full multi-locus sequence typing using the MLST *E. coli* typing system, primers and conditions described at the university of Warwick http://mlst.warwick.ac.uk/mlst/dbs/Ecoli.

Supplemental Results

Initial Species Identification

Initial species identification was by colony color on brilliance agar plates. Swabs from each site were plated out on individual plates. Clear colony types >100 were found on all plates, indicative of carbapenem resistant non-fermentative bacteria being found at all sites. Red colonies indicative of *E. coli* were found on 11 plates varying in number from 1 or 2 colonies to 50-80 per plate. Dark blue colonies suggestive of *K. pneumoniae* were found on 19 plates and varied in number from 1 or 2 to more than 100 per plate.

Comparison of *E. coli* Phylogeny Methods

The 2 locus clonal EXPEC typing method correctly identified all isolates belonging to ST101 and ST405 whereas the Clermont method was inaccurate for many of the strains analyzed placing ST648 and ST405 (phylogroup D) as B2 as well as half of the ST101 (phylogroup B1) as phylogroup E (Technical Appendix Table 2). Interestingly, all ST648 phylogroup D *E. coli* strains were *fim*H negative and *fumC* allele 4 (Technical Appendix Table 2). Only a single phylogroup B1 strain was present with this 2 locus designation in the database and so this system incorrectly identified this ST648 as phylogroup B1 instead of D.

Resistance Profile of *bla*$_{NDM-1}$ Bacterial Species

Overall the bacterial isolates displayed antibiotic resistance profiles that are very similar to clinical isolates that carry *bla*$_{NDM-1}$ (Technical Appendix Table 3). The *E. coli* and *K. pneumoniae* isolates were resistant to all β-lactams with the single exception of one *K. pneumoniae* strain that was sensitive to Aztreonam. They were also resistant to all clinical aminoglycosides and only sensitive to fosfomycin, tigecycline and colistin reflecting the additional presence of 16s RNA methylases in all strains classifying the majority of these isolates as XDR. In general, the MICs to meropenem were higher than those to imipenem, validating the inclusion of meropenem in the initial screening. The various *Acinetobacter* spp. and *Citrobacters* were generally less resistant, several being sensitive to aminoglycosides and occasionally ciprofloxacin, the *Acinetobacters* were additionally generally sensitive to rifampicin.

Technical Appendix Table 1. Isolate list/NDM plasmid profile*

Area/site, isolate no.	Species	NDM plasmid size, kb,
MOT/1.1		
1	*Citrobacter freundii*	50, 250
177	*C. freundii*	50, 250
MOT/1.2		
5	*C. freundii*	50, 250
MOT/1.3		
13	*C. freundii*	50, 250
14	*C. freundii*	50, 250
16	*C. freundii*	50, 250
179	*C. freundii*	50, 250
180	*C. freundii*	50, 250
ARG/2.1		
17	*Escherichia coli*	150
18	*E. coli*	150
19	*E. coli*	150
20	*E. coli*	150
23	*E. coli*	150
24	*E. coli*	150
25	*E. coli*	150
83	*Acinetobacter tandoii*	240
84	*A. tandoii*	ND
86	*Klebsiella pneumoniae*	50,120,180,250
87	*K. pneumoniae*	100,180,250
89	*K. pneumoniae*	100
93	*Acinetobacter genomospecies*	240
94	*A. genomospecies*	ND
95	*K. pneumoniae*	100
97	*K. pneumoniae*	100
98	*Acinetobacter towneri*	ND
ARG/2.2		
27	*E. coli*	150
28	*E. coli*	150
29	*E. coli*	150
30	*E. coli*	150
31	*E. coli*	150
32	*E. coli*	150
33	*E. coli*	150
34	*E. coli*	150
ARG/2.6		
35	*K. pneumoniae*	100
36	*K. pneumoniae*	100
ARG/2.7		
43	*E. coli*	100
44	*E. coli*	100
45	*E. coli*	100
46	*E. coli*	100
47	*K. pneumoniae*	100
49	*K. pneumoniae*	100
50	*K. pneumoniae*	100
51	*K. pneumoniae*	50, 250
54	*E. coli*	100
59	*K. pneumoniae*	145,160
182	*K. pneumoniae*	145,160
183	*Pseudomonas putida*	ND
184	*K. pneumoniae*	50, 250
185	*Pseudomonas alcaligenes*	ND
ARG/2.8		
186	*K. pneumoniae*	145,160
69	*K. pneumoniae*	145,160
70	*K. pneumoniae*	145,160
ARG/2.9		
73	*K. pneumoniae*	120
80	*K. pneumoniae*	120
GUL/3.1		
99	*A. towneri*	50
100	*A. towneri*	ND
GUL/3.2		

Area/site, isolate no.	Species	NDM plasmid size, kb,
103	*Pseudomonas* spp.	ND
105	*P. putida*	ND
107	*A. tandoii*	50
108	*A. tandoii*	ND
GUL/3.3		
109	*Pseudomonas* spp.	ND
112	*P. putida*	ND
116	*P. putida*	ND
118	*P. putida*	ND
120	*Pseudomonas otitidis*	ND
GUL/3.4		
121	*P. putida*	ND
125	*P. otitidis*	ND
128	*P. putida*	ND
GUL/3.5		
138	*P. otitidis*	ND
GUL/3.6		
144	*P. putida*	ND
146	*P. otitidis*	ND
147	*Pseudomonas monteilii*	ND
ADA/4.3		
151	*K. pneumoniae*	120
153	*K. pneumoniae*	120
155	*A. towneri*	ND
156	*K. pneumoniae*	100, 350
158	*K. pneumoniae*	100, 350
159	*P. aeruginosa*	ND
160	*A. towneri*	ND
ADA/4.4		
161	*Acinetobacter baumannii*	250, 450
162	*Pseudomonas mendocina*	ND
163	*A. genomospecies*	280
165	*A. genomospecies*	280
166	*P. mendocina*	ND
167	*K. pneumoniae*	ND
170	*A. genomospecies*	ND
174	*A. genomospecies*	ND
175	*A. genomospecies*	ND
176	*A. genomospecies*	ND
ADA/4.5		
188	*E. coli*	150
189	*E. coli*	150
190	*K. pneumoniae*	45, 150, 250
191	*E. coli*	130
192	*E. coli*	130
193	*E. coli*	130
194	*E. coli*	130
195	*E. coli*	130
197	*K. pneumoniae*	45, 150, 250
199	*K. pneumoniae*	45, 150, 250
200	*E. coli*	270
201	*K. pneumoniae*	100, 350
202	*Pseudomonas monteilii*	ND
204	*P. putida*	ND
205	*K. pneumoniae*	100, 350
ADA/4.6		
207	*K. pneumoniae*	50, 100, 250
208	*E. coli*	100
209	*K. pneumoniae*	100, 300
210	*K. pneumoniae*	50, 120, 140
213	*E. coli*	140
214	*E. coli*	140
211	*E. coli*	130
212	*K. pneumoniae*	50, 120, 140
216	*K. pneumoniae*	50, 120, 140
217	*K. pneumoniae*	120, 200
218	*K. pneumoniae*	120, 200

Area/site, isolate no.	Species	NDM plasmid size, kb,
219	*K. pneumoniae*	80, 130, 150
220	*P. putida*	ND
ADA/4.9		
221	*E. coli*	130
222	*E. coli*	130
225	*P. putida*	ND
226	*P. putida*	ND
227	*P. putida*	ND
228	*C. freundii*	50, 250
229	*K. pneumoniae*	80, 130, 150
230	*K. pneumoniae*	150, 170, 300
231	*K. pneumoniae*	150, 170, 300
233	*K. pneumoniae*	50, 150, 200
236	*P. mendocina*	ND
ADA/4.10		
237	*Pseudomonas fulva*	ND
238	*Pseudomonas aeruginosa*	ND
239	*E. coli*	130
241	*K. pneumoniae*	50, 150, 200
242	*P. aeruginosa*	ND
243	*C. freundii*	50, 250
245	*Aeromonas caviae*	ND
249	*P. aeruginosa*	ND
ADA/4.11		
250	*E. coli*	130
251	*E. coli*	130
252	*E. coli*	130
253	*E. coli*	130
254	*E. coli*	130
255	*E. coli*	130
256	*E. coli*	130
257	*E. coli*	130
258	*K. pneumoniae*	110
259	*K. pneumoniae*	110
260	*K. pneumoniae*	150
261	*K. pneumoniae*	150
262	*A. baumannii*	ND
264	*K. pneumoniae*	150
265	*K. pneumoniae*	150
266	*K. pneumoniae*	150
267	*K. pneumoniae*	120
ADA/4.12		
268	*E. coli*	130
269	*E. coli*	130
271	*E. coli*	140
273	*E. coli*	140
274	*E. coli*	130
275	*E. coli*	130
276	*K. pneumoniae*	120
277	*K. pneumoniae*	120
278	*K. pneumoniae*	120
279	*K. pneumoniae*	120
280	*K. pneumoniae*	120
281	*K. pneumoniae*	120
282	*K. pneumoniae*	ND
283	*K. pneumoniae*	ND

Area/site, isolate no.	Species	NDM plasmid size, kb,
LAL/5.1		
342	*K. pneumoniae*	ND
343	*K. pneumoniae*	ND
344	*K. pneumoniae*	ND
345	*K. pneumoniae*	ND
346	*E. coli*	130
347	*E. coli*	130
348	*E. coli*	120
349	*E. coli*	140
LAL/5.2		
352	*P. putida*	ND
355	*P. mendocina*	ND
358	*K. pneumoniae*	ND
360	*P. mendocina*	ND
361	*P. oleovorans*	ND
362	*P. oleovorans*	ND
LAL/5.7		
363	*P. oleovorans*	ND
364	*Pseudomonas pseudoalcaligenes*	ND
LAL/5.8		
367	*P. fulva*	ND
368	*P. fulva*	ND
369	ND	ND
370	ND	ND
0-P/6.2		
287	*P. monteilii*	ND
288	*P. putida*	ND
289	*P. putida*	ND
291	*P. putida*	ND
292	*P. putida*	ND
0-P/6.3		
293	*K. pneumoniae*	ND
294	*K. pneumoniae*	ND
295	*P. putida*	ND
296	*K. pneumoniae*	ND
297	*K. pneumoniae*	ND
298	*K. pneumoniae*	ND
299	*P. otitidis*	ND
300	*P. otitidis*	ND
301	*P. otitidis*	ND
303	*P. putida*	ND
304	*P. putida*	ND
305	*P. putida*	ND
306	ND	ND
0-P/6.4		
307	*K. pneumoniae*	ND
308	*K. pneumoniae*	ND
309	ND	ND
310	*P. mendocina*	ND
311	ND	ND
312	ND	ND
313	*P. otitidis*	ND

Area/site, isolate no.	Species	NDM plasmid size, kb,
314	ND	ND
315	*P. putida*	ND
316	*P. putida*	ND
0-P/6.8		
323	*K. pneumoniae*	ND
324	*K. pneumoniae*	ND
325	*K. pneumoniae*	ND
326	*K. pneumoniae*	ND
327	*K. pneumoniae*	ND
328	ND	ND
330	*K. pneumoniae*	ND
331	ND	ND
332	ND	ND
333	ND	ND
335	*Pseudomonas oleovorans*	ND
0-P/6.9		
336	*P. otitidis*	ND
337	*P. otitidis*	ND
338	ND	ND
339	ND	ND
MOH/7.5		
340	ND	ND
341	ND	ND
371	*P. mendocina*	ND
372	*P. pseudoalcaligenes*	ND
MOH/7.6		
374	*P. oleovorans*	ND
375	*P. mendocina*	ND
376	*P. oleovorans*	ND
377	*P. pseudoalcaligenes*	ND
378	*P. oleovorans*	ND

*Arg, Argargoan;Gul, gulshan; Lal, Lalbag; Moh, Mohammad pur; Mot, Motijheel; ND, not determined; NDM, New Delhi metallo-β-lactamase; 0-p, 0 point (Dhaka city regions).

Technical Appendix Table 2. Phylogenetic analysis of NDM-positive *Escherichia coli* from the environment, Bangladesh*

				Clermont strain typing analysis				2 -loci typing analysis			
Strain no.	NDM	Site	Area in Bangladesh	*chu*A PCR	*yja*A PCR	*tsp*E PCR	Apparent designation	*fim*H allele	*fum*C allele	Designation by 2 loci	MLST, true designation
17	+	2.1	Argargoan		+	+	B2	258	41	B1	B1-ST101
18	NDM-3	2.1	Argargoan		+		E	258	41	B1	B1-ST101
19	+	2.1	Argargoan		+		E	258	41	B1	B1-ST101
20	+	2.1	Argargoan		+		E	258	41	B1	B1-ST101
23	+	2.1	Argargoan		+		E	258	41	B1	B1-ST101
24	NDM-3	2.1	Argargoan		+	+	B2	258	41	B1	B1-ST101
25	+	2.1	Argargoan		+		E	258	41	B1	B1-ST101
27	+	2.2	Argargoan		+		E	258	41	B1	B1-ST101
28	NDM-3	2.2	Argargoan		+	+	E	258	41	B1	B1-ST101
29	+	2.2	Argargoan		+		E	258	41	B1	B1-ST101
30	+	2.2	Argargoan		+	+	B2	258	41	B1	B1-ST101
31	+	2.2	Argargoan		+	+	B2	258	41	B1	B1-ST101
32	+	2.2	Argargoan		+		E	258	41	B1	B1-ST101
33	+	2.2	Argargoan		+		E	258	41	B1	B1-ST101
34	+	2.2	Argargoan		+	+	B2	258	41	B1	B1-ST101
43	NDM-1	2.7	Argargoan	+	+	+	B2	37	27	D	D-ST405
44	+	2.7	Argargoan	+	+	+	B2	37	27	D	D-ST405
45	+	2.7	Argargoan	+	+	+	B2	37	27	D	D-ST405
46	+	2.7	Argargoan		+	+	B2	37	27	D	D-ST405
54	NDM-1	2.7	Argargoan	+	+	+	B2	37	27	D	D-ST405
188	+	4.5	Adabor	+		+	B2	Null	4	B1	D-ST648
189	+	4.5	Adabor	+		+	B2	Null	4	B1	D-ST648
191	+	4.5	Adabor	+		+	B2	Null	4	B1	D-ST648
192	NDM-4	4.5	Adabor	+		+	B2	Null	4	B1	D-ST648
193	+	4.5	Adabor	+		+	B2	Null	4	B1	D-ST648
194	+	4.5	Adabor	+		+	B2	Null	4	B1	D-ST648
195	+	4.5	Adabor	+	+	+	B2	Null	4	B1	D-ST648

200	+	4.5	Adabor		+	+	B2	258	41	B1	B1-ST101
208	+	4.6	Adabor	+		+	B2	Null	4	B1	D-ST648
211	+	4.6	Adabor	+		+	B2	Null	4	B1	D-ST648
213	+	4.6	Adabor	+	+	+	B2	Null	4	B1	D-ST648
214	+	4.6	Adabor	+		+	B2	Null	4	B1	D-ST648
221	NDM-1	4.9	Adabor		+	+	B2	258	41	B1	B1-ST101
222	+	4.9	Adabor		+		E	258	41	B1	B1-ST101
239	+	4.10	Adabor		+	+	B2	258	41	B1	B1-ST101
250	+	4.11	Adabor	+	+	+	B2	Null	4	B1	D-ST648
251	+	4.11	Adabor	+	+	+	B2	Null	4	B1	D-ST648
252	+	4.11	Adabor	+	+	+	B2	Null	4	B1	D-ST648
253	+	4.11	Adabor	+	+	+	B2	Null	4	B1	D-ST648
254	NDM-1	4.11	Adabor	+	+	+	B2	Null	4	B1	D-ST648
255	+	4.11	Adabor	+	+	+	B2	Null	4	B1	D-ST648
256	+	4.11	Adabor	+	+	+	B2	Null	4	B1	D-ST648
257	+	4.11	Adabor	+	+	+	B2	Null	4	B1	D-ST648
268	+	4.12	Adabor	+	+	+	B2	Null	4	B1	D-ST648
269	+	4.12	Adabor	+	+	+	B2	Null	4	B1	D-ST648
271	NDM-1	4.12	Adabor			+	*E. albertii*	258	41	B1	B1-ST101
273	+	4.12	Adabor	+	+	+	B2	Null	4	B1	D-ST648
274	+	4.12	Adabor				?	Null	4	B1	D-ST648
275	+	4.12	Adabor	+	+	+	B2	Null	4	B1	D-ST648
346	+	5.1	Lalbag	+	+	+	B2	Null	4	B1	D-ST648
347	NDM-1	5.1	Lalbag	+	+	+	B2	Null	4	B1	D-ST648
348	+	5.1	Lalbag	+	+	+	B2	Null	4	B1	D-ST648
349	+	5.1	Lalbag	+	+	+	B2	Null	4	B1	D-ST648

*NDM, New Delhi-metallo-β-lactamase; MLST, multilocus sequence typing.

Technical Appendix Table 3. MICs of various antibiotics to a subset of individual NDM encoding environmental bacteria*

Genus sp., ST,	Antimicrobial drug																			
strain no.	IMP	MEM	ERT	PIP	AMX/CLA	PIP/TAZ	AMP	CAZ/CLA	CTX/CLA	FEP/CLA	ATM	AMK	TOB	GEN	CIP	TGC	FOF	NIT	CST	RIF
Escherichia coli																				
ST101																				
18	4	12	>32	>256	>256	>256	>256	>32/>4	>16/>1	>16/>4	>256	>256	>256	ND	>32	0.19	2	>512	1	ND
24	12	>32	>32	>256	>256	>256	>256	>32/>4	>16/>1	>16/>4	>256	>256	>256	ND	>32	0.25	1.5	>512	0.25	ND
25	4	16	>32	>256	>256	>256	>256	>32/>4	>16/>1	>16/>4	>256	>256	>256	ND	>32	0.5	24	192	1	ND
28	>32	>32	>32	>256	>256	>256	>256	>32/>4	>16/>1	>16/>4	>256	>256	>256	ND	>32	0.25	1.5	>512	0.75	ND
221	6	6	12	>32	>256	>256	>256	>32/>4	>16/>1	>16/>4	>256	>256	>256	ND	>32	0.38	2	>512	1	ND
ST648																				
40	6	12	>32	>256	>256	>256	>256	>32/>4	>16/>1	>16/>4	>256	>256	>256	ND	>32	0.19	2	48	1	ND
192	12	>32	>32	>256	>256	>256	>256	>32/>4	>16/>1	>16/>4	>256	>256	>256	ND	>32	0.38	1	192	1	ND
346	4	>32	>32	>256	>256	>256	>256	>32/>4	>16/>1	>16/>4	>256	>256	>256	ND	>32	0.25	1	>256	1	ND
ST405																				
43	8	>32	>32	>256	>256	>256	>256	>32/>4	>16/>1	>16/>4	>256	>256	>256	ND	>32	0.38	3	48	1	ND
54	6	12	>32	>256	>256	>256	>256	>32/>4	>16/>1	>16/>4	>256	>256	>256	ND	>32	0.19	2	48	1	ND
Citrobacter freundii																				
1	4	6	6	>256	>256	>256	>256	>32/>4	>16/>1	>16/>4	>256	>256	12	0.5	>32	1	2	>512	0.75	32
5	2	3	>32	>256	>256	>256	>256	>32/>4	>16/>1	>16/>4	>256	>256	12	0.38	>32	0.75	3	16	1	32
172	4	16	>32	>256	>256	>256	>256	>32/>4	>16/>1	>16/>4	>256	>256	12	0.38	>32	1	2	24	0.75	16
228	2	4	8	>256	>256	>256	>256	>32/>4	>16/>1	>16/>4	>256	>256	12	1.5	3	1	48	16	0.5	24
243	6	6	6	>256	>256	>256	>256	>32/>4	>16/>1	>16/>4	>256	>256	12	0.75	3	1	2	16	1	24
Klebsiella pneumoniae																				
35	8	>32	>32	>256	>256	>256	>256	>32/>4	>16/>1	>16/>4	>256	>256	>256	ND	>32	0.5	16	>512	0.38	ND
49	>32	>32	>32	>256	>256	>256	>256	>32/>4	>16/>1	>16/>4	>256	>256	>256	ND	>32	0.75	12	384	0.5	ND
70	6	6	>32	>256	>256	>256	>256	>32/>4	>16/>1	>16/>4	>256	>256	>256	ND	>32	0.5	12	>512	1	ND
86	8	6	>32	>256	>256	>256	>256	>32/>4	>16/>1	>16/>4	>256	>256	>256	ND	>32	0.5	12	>512	0.75	ND
151	>32	12	>32	>256	>256	>256	>256	>32/>4	>16/>1	>16/>4	>256	0.25	>256	ND	>32	64	16	>512	2	ND
182	8	24	>32	>256	>256	>256	>256	>32/>4	>16/>1	>16/>4	>256	>256	>256	ND	>32	0.5	12	>512	1	ND
201	3	8	>32	>256	>256	>256	>256	>32/>4	>16/>1	>16/>4	>256	>256	>256	ND	6	0.5	6	96	0.75	ND
297	>32	16	>32	>256	>256	>256	>256	>32/>4	>16/>1	>16/>4	>256	>256	>256	ND	12	1	4	384	0.5	ND
323	6	>32	>32	>256	>256	>256	>256	>32/>4	>16/>1	>16/>4	>256	>256	>256	ND	>32	0.75	16	>512	0.38	ND
325	12	>32	>32	>256	>256	>256	>256	>32/>4	>16/>1	>16/>4	>256	>256	>256	ND	>32	0.75	16	>512	0.75	ND
Acinetobacter towneri																				
83	32	16	>32	64	>256	32	>256	>32/>4	>16/>1	>16/>4	>256	>256	3	48	0.25	0.19	12	12	0.5	1.5
93	>32	>32	>32	>256	>256	>256	>256	>32/>4	>16/>1	>16/>4	>256	>256	256	192	>32	0.5	32	32	0.75	16
Actinobacillus genomosp.																				
98	>32	>32	>32	96	>256	48	>256	>32/>4	>16/>1	>16/>4	>256	>256	0.38	0.19	0.5	0.094	8	8	0.75	1.5
155	>32	>32	>32	96	>256	24	>256	>32/>4	>16/>1	>16/>4	>256	>256	1.5	24	32	0.125	8	8	0.5	1.5
Acinetobacter tandoii																				
161	>32	>32	>32	>256	>256	64	>256	>32/>4	>16/>1	>16/>4	>256	>256	12	192	>32	0.19	32	32	0.75	4
A. baumannii																				
174	>32	>32	>32	96	>256	32	>256	>32/>4	>16/>1	>16/>4	>256	>256	6	128	0.19	0.125	32	32	0.75	2
262	32	16	>32	>256	>256	>256	>256	>32/>4	>16/>1	>16/>4	>256	>256	256	256	>32	0.75	64	64	1	6

*AMK, amikacin; AMP, ampicillin; AMX, amoxicillin; ATM, aztreonam; CAZ, Ceftazidime; CIP, ciprofloxacin; CLA, clavulanic acid; CST, colistin; CTX, cefotaxime; ERT, ertapenem; FEP, cefipime; FOF, fosfomycin; GEN, gentamicin; IMP, imipenem; MEM, meropenem; ND, not determined; NDM, New Delhi-metallo-β-lactamase; NIT, nitrofurantoin; PIP, piperacillin; RIF, rifampin; ST, sequence type; TAZ, tazobactam; TGC, tigecycline; TOB, tobramycin

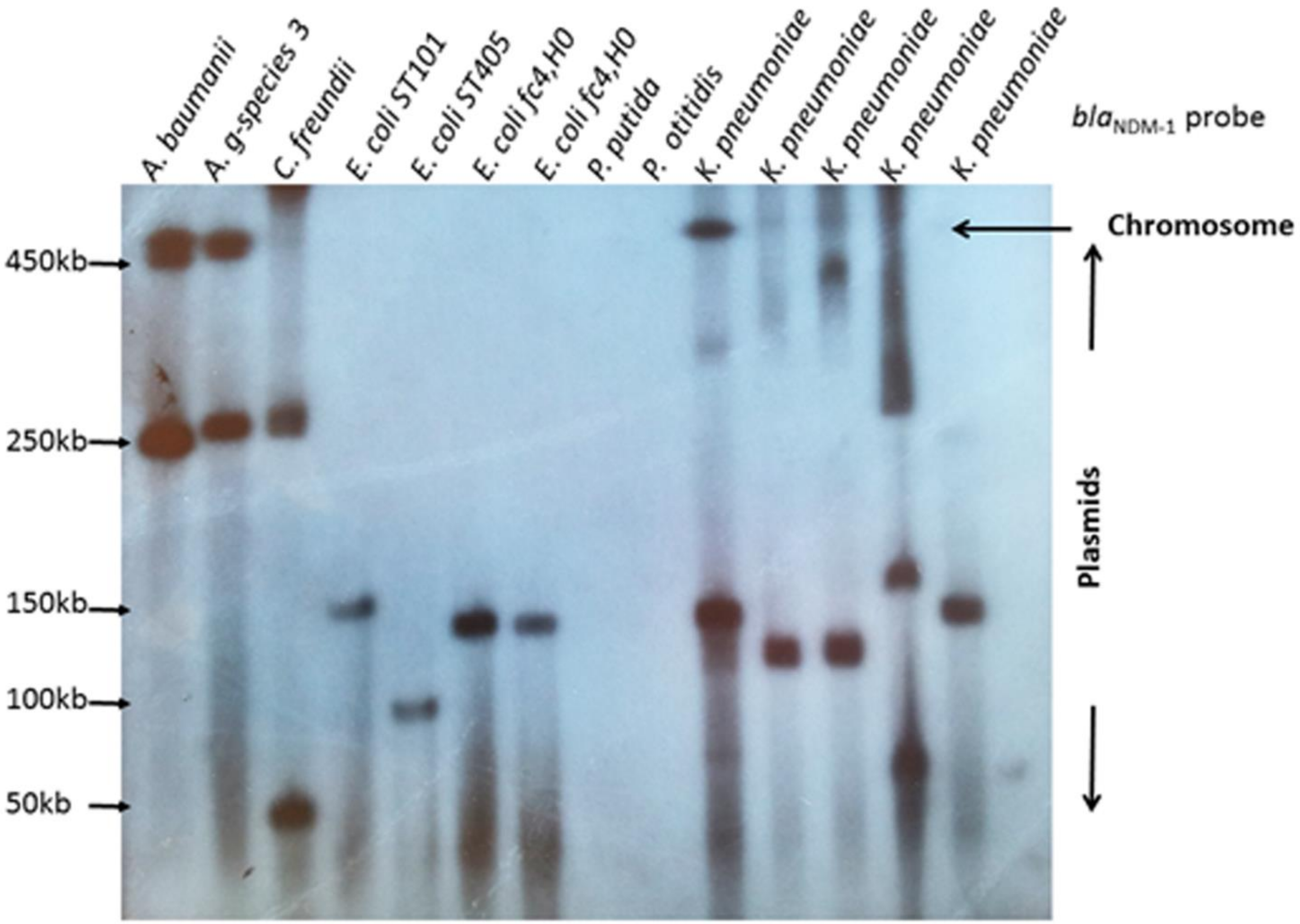

Technical Appendix Figure. Genomic location of *bla*$_{NDM-1}$ in environmental bacteria from Dhaka, Bangladesh. Pulsed field gel electrophoresis of macro DNA of a subset of *bla*$_{NDM-1}$–harboring strains collected from environmental waters in Dhaka. The gel was probed with a radio-labeled *bla*$_{NDM-1}$ probe and detected by using photographic film. Samples were run against a concatenated λ molecular size standard.
